# Comparison of the Correlations of Microbial Community and Volatile Compounds between Pit-Mud and Fermented Grains of Compound-Flavor Baijiu

**DOI:** 10.3390/foods13020203

**Published:** 2024-01-08

**Authors:** Wei Cheng, Xuefeng Chen, Xijia Xue, Wei Lan, Huawei Zeng, Ruilong Li, Tianquan Pan, Na Li, Zilu Gong, Hongwen Yang

**Affiliations:** 1 School of Food Science and Engineering, Shaanxi University of Science and Technology, Xi’an 710021, China; chengwei130@jzz.cn; 2 Technology Center of Enterprise, Anhui Jinzhongzi Distillery Co., Ltd., Fuyang 236023, China; xuexijia@jzz.cn (X.X.); pantianquan@jzz.cn (T.P.); lina1036@jzz.cn (N.L.); gongzilu@jzz.cn (Z.G.); yanghongwen@jzz.cn (H.Y.); 3 School of Biology and Food Engineering, Fuyang Normal University, Fuyang 236037, China; pennylrl@163.com; 4 School of Life Sciences, Huaibei Normal University, Huaibei 235000, China; huaweizeng@163.com

**Keywords:** compound-flavor baijiu (CFB), fermented grains (FG), microbial community, pit mud (PM), volatile compounds

## Abstract

The microbial composition and volatile components of fermented grains (FG) and pit mud (PM) are crucial for the quality and flavor of compound-flavor baijiu (CFB). The physicochemical indices, culturable microorganisms, microbial communities, and volatile components of FG and PM were analyzed and correlated in our research. Considering FG and PM, amplicon sequencing was used to analyze the microbial community and the volatile components were detected by headspace solid-phase microextraction-gas chromatography–mass spectrometry (HS-SPME). For FG, redundancy analysis and correlation perfume Circos were used to clarify the correlations between the dominant microbial community and volatile components. The results showed that *Aspergillus*, *Pichia,* and *Rhizopus* were the main fungal microflora in FG and PM, whereas *Lactobacillus* and *Bacillus* were the dominant bacteria in FG, and *Methanosarcina* and *Clostridium sensu stricto 12* were the dominant bacteria in the PM. The microbial community and volatile compounds in the CB sampled from the bottom layers of the FG were greatly affected by those in the PM. There were 32 common volatile components in CB and PM. For FG, most of the volatile components were highly correlated with *Lactobacillus*, *Bacillus*, *Aspergillus*, *Pichia,* and *Monascus*, which includes alcohols, acids and esters. This study reveals correlations between microbial composition, volatile components, and the interplay of FG and PM, which are conducive to optimizing the fermentation process and improving the quality of CFB base.

## 1. Introduction

Baijiu (Chinese Baijiu) is one of the six major distilled spirits in the world. It is usually obtained from a mixture of sorghum, barley, wheat and corn or sorghum alone as raw materials through fermentation, distillation, and storage. Saccharification starter cultures, specifically Daqu, Xiaoqu, and Fuqu, orchestrate fermentation through intricate enzymatic chemical reactions, biochemical processes, and microbial metabolic activities [1,2,3]. The essence of compound-flavor baijiu (CFB) often lies in its distinctive and intricate aromatic profiles, rooted in four fundamental flavor styles [4]. This study introduces CFB fermentation techniques that harmoniously amalgamate the craftsmanship of sauce-flavored baijiu, strong-flavored baijiu, and sesame-flavored baijiu. The amalgamated process undergoes solid-state fermentation over a span of approximately 60–80 days, and the fermentation vessel under scrutiny, its construction features a pit wall hewn from slate, with the bottom fashioned from pit mud (PM) [5]. The course of fermentation is a harmonious symphony wherein grains, microorganisms, metabolites, and enzymes converge, instigating an array of biochemical reactions that intricately influence the character and flavor profile of Baijiu [4,6]. Furthermore, the intricate interplay of microbial communities and metabolites residing in the PM wield a profound sway over the caliber of fermented grains (FG) [7,8]. This, in turn, stands as one of the foremost determinants in shaping the quality benchmarks of CFB.

In the realm of solid-state Baijiu fermentation, comprehending the diversity and metabolic attributes of microbial communities holds paramount significance. This understanding aids in elucidating the intricate connections between microbial composition and flavor metabolism, subsequently shedding light on the nuanced facets of Baijiu fermentation conditions [9,10]. Guan et al. embarked on an exploration of a robust baijiu’s upper fermented grains (FG) via high-throughput sequencing, identifying Firmicutes, Actinobacteriota, Proteobacteria, Ascomycota, and Basidiomycota as the predominant phyla [9]. Through the prism of typical correspondence analysis (CCA) and correlation network, the intricate interplay between microbial communities, physicochemical contexts, and flavor compounds were unveiled. Noteworthy correlations emerged, with temperature, oxygen levels, and acidity exhibiting strong correlations with microbial communities. Simultaneously, an array of flavor compounds demonstrated positive associations with entities such as *Aspergillus Calmette*, *Delftia*, *Caproiciproducens*, *Thermoactinomyces*, *Hydrogenispora*, *Issatchenkia Bacillus*, and *Aspergillus* [9]. Yang et al. embarked on a deep exploration of stacking fermentation as a pivotal phase of microbial amplification and enrichment within the Moutai-flavored baijiu brewing process, which exerts a profound sway over wine quality [11]. Over 1–6 rounds of stacked fermentation for Moutai-flavored baijiu, their investigation delved into the spatial and temporal congruities and disparities in microbial community structure and succession. Revelations surfaced, underscoring heightened microbial diversity and richness in the core of the pile compared to its surface. A shift in the dominant genus from *Lactobacillus* to *Acetobacter* was observed, coupled with a gradual replacement of the leading fungal genus by *Candida albicans*. It’s noteworthy that certain microorganisms (such as *Acetobacillus* and *Thermus thermophilus*) couldn’t assert dominance over the pile surface and core [11].

The microbial composition and volatile constituents residing within FG and PM wield a pivotal sway over the nuanced flavor intricacies and overall quality of CFB. A plethora of studies have extensively documented the physicochemical indices, microbial composition, and volatile components implicated in the fermentation process of FG [10,12]. Nonetheless, investigations into the intricate interplay among physicochemical indices, microbial composition, and volatile constituents within FG at varying depths within fermentation cellars remain scarce [13]. Additionally, the precise correlations interlinking the microbial composition and volatile components of both FG and PM remain elusive. Furthermore, a comprehensive exploration into the ramifications stemming from the microbial composition and volatile components within PM upon FG samples extracted from distinct layers necessitates further scholarly inquiry.

This study undertook an examination of the microbial composition and volatile constituents found within PM and FG sourced from diverse strata within the fermentation cellars. Moreover, correlation analysis of the correlations interconnecting these indicators was conducted to reveal the intricate interplay among physicochemical indices, microbial composition, and volatile constituents within FG at varying depth, which are conducive to optimizing the fermentation process and improving the quality of CFB base.

## 2. Materials and Methods

### 2.1. Sample Collection

FG and PM samples were collected from the CFB brewing pit of Anhui Jin-zhongzi Distillery Co., Ltd. (Fuyang, China) After fermentation (65 days), FG from three layers of the fermentation cellar (the upper, middle, and bottom layers of fermentation cellar) were sampled from five points in each layer (Figure S1), mixed, and named as CA, CM, and CB, respectively. PM was collected from the bottom of the fermentation cellar, sampled from five points on the diagonal, mixed (Figure S1), and named as CP. Three parallel FG and PM samples were collected randomly, divided into two parts, and packed in two separate bags. One bag, stored at 4 °C, was used for the detection of physicochemical parameter and plate counting of culturable microorganisms; the other bag, stored at −80 °C, was used for the extraction of total DNA.

### 2.2. Determination of Physicochemical Indexes of FG and PM

For FG, drying the samples at 105 °C until a constant weight was observed to detect the moisture content. As the previously described [14,15], the total acidity, starch content, and reducing sugar content of FG were determined. According to the method described by Zhang et al. [16], the PM physicochemical indices were determined. The atmospheric pressure drying method was used to detect the moisture content. After extracting PM with water, the pH value was measured using a pH meter, and ammonia nitrogen was determined by UV–visible spectrophotometry. In addition, available phosphorus (available P), available potassium (available K), calcium, and humus con-tents were determined using atomic absorption spectrometry.

### 2.3. Plate Counting of Culturable Microorganisms in FG and PM

Taken FG sample (25 g) and placed in a conical flask, adding 225 mL distilled water, and the mixture was shaken at 120 rpm for 30 min. Then, 1 mL of the sus-pension was pipetted and diluted by 10-times gradients to an appropriate concentration [17]. Bacteria were cultured in Luria–Bertani medium (LB) at 37 °C for two days [18]; molds were cultured in Czapek Dox medium (CD) [17], containing 100 µg/mL ampicillin, at 30 °C for three days; yeast were cultured in yeast extract peptone dextrose medium (YPD), and 100 µg/mL ampicillin was contained, at 30 °C for three days [19]; all microbial colonies were counted separately. For PM, the incubation carried out under the anaerobic condition to counting the culturable microorganisms [14,16].

### 2.4. High Throughput Sequencing

All samples were pretreated with sterile phosphate-buffered saline (PBS, 0.1 mol/L), then centrifuged at 30 g for 10 min, and the supernatant was then centrifuged again at 10,000 rpm for 10 min. The precipitated cells obtained were ground with liquid nitrogen and used for DNA extraction using the E.Z.N.A. Soil DNA Kit (Omega Bio-tek, Norcross, GA, USA), according to the manufacturer’s instructions [20,21]. According to the selection of the sequencing region (Phusion^®^ High-Fidelity PCR Master Mix with GC Buffer: New England Biolabs, Ipswich, MA, USA, New England Biolabs (Beijing) LTD., Beijing, China), diluted genomic DNA was used as template and specific primers with barcodes were used; high-efficiency HiFi enzymes were used for PCR to ensure amplification efficiency and accuracy. For bacteria, 515F (5′–GTGYCAGCMGCCGCGGTAA–3′) was used as forward primer and 806R (5′–GGACTACNVGGGTWTCTAAT–3′) amplified 16S V3–V4 domain was used as do-main reverse primer; for fungi, ITS5-1737F (5′–GGAAGTAAAAGTCGTAACAAGG–3′) and ITS2-2043R (5′–GCTGCGTTCTTCATCGATGC–3′) amplified the ITS 1 sequence region.

The QuantiFluor-ST Blue Fluorescence Quantitative System (Promega, Madison, WI, USA) was used to assess the concentration of the polymerase chain reaction products. Finally, the MiSeq Benchtop Sequencer (2×300 bp; Illumina MiSeqPE300, San Diego, CA, USA) was used to sequence the total genomic DNA by Bioyigene Biotechnology Co., Ltd. (Wuhan, China).

### 2.5. Analysis of Volatile Components by HS-SPME-GC-MS

#### 2.5.1. Analysis of Volatile Components in FG

Pretreatment of FG samples were performed as described previously [14]. Then, 10 g of sample was weighed and mixed with 1% CaCl_2_, boiled in 20 mL ultra-pure water, and soaked overnight at 4 °C. After ultrasound in ice water bath for 30 min, the supernatant was separated by centrifugation at 10,000 rpm at 4 °C for 20 min. Then, 5 mL of the supernatant was placed in a 20 mL headspace bottle with 3 g of NaCl and a quantitative internal standard, amyl acetate (20 µL, 0.3612 µg/L).

A three-phase extraction head (DVB/CAR/PDMS, 50/30 µm) was used during extraction at initial temperature 50 °C, preheating for 5 min, extrac-tion for 45 min, and desorption for 5 min. For GC, a DB-FFAP (Nitro-terephthalic acid modified polyethylene glycol) chromatographic column (60 m × 0.25 mm × 0.25 µm, Agilent Technologies, Santa Clara, CA, USA) was used with the following gradient condition: initial temperature 50 °C for 2 min, then raising the temperature to 230 °C at 6 °C/min, held for 15 min. The temperatures of the inlet and detector were kept at 250 °C, use the high purity helium (He) as the carrier gas, and the flow rate was maintained at 2 mL/min, not shunt. For MS, use 250 °C as the junction temperature, 150 °C as the quadrupole temperature, and 35–350 amu as the scanning range.

#### 2.5.2. Analysis of Volatile Components in PM

Pretreatment of PM samples were performed as described previously [15,22]. Ap-proximately 1 g of PM was weighed and added into a 20 mL headspace bottle, with the quantitative internal standard, sec-octanol (10 µL, 0.029949 g/L). The sample was balanced at 50 °C for 15 min, inserted into a fiber extractor for adsorption and extracted for 45 min, and imported into GC sampling mouth for thermal analysis for 5 min; sample was then subjected to GC–MS to analyze the volatile components. A three-phase extraction head (DVB/CAR/PDMS, 50/30 µm) was used during extraction at initial temperature 50 °C, preheating for 5 min, extraction for 45 min, and desorption for 5 min.

For GC, a DB-FFAP chromatographic column (60 m × 0.25 mm × 0.25 µm, Agilent Technologies, Santa Clara, CA, USA) and the following gradient conditions were used: inlet temperature 250 °C, carrier gas (He) flow rate 1 mL/min, non-shunt mode sampling, and mass spectrum connection line temperature 240 °C. The heating procedure was as follows: initial column temperature was maintained at 40 °C for 2 min, increased to 130 °C at 3 °C/min, increased to 200 °C at 6 °C/min, and finally increased to 230 °C at 8 °C/min, kept for 8 min. For MS, electron bombardment energy was 70 eV, ion source temperature was 230 °C, four-stage rod temperature was 150 °C, and a scanning of 35–450 amu was used.

### 2.6. Data Processing and Statistic Analysis

The processing of raw sequencing data was done as follows: after the completion of high-throughput sequencing, the quality of the sequencing data was optimized by QIIME2 (2019.4) software (Illumina, San Diego, CA, USA), and a sequence similarity greater than or equal to 97% was classified into the same operational taxonomic unit (OTU). To obtain taxonomic information, taken all sequences to compare with the Silva (Silva_138.1) database. The community composition map and heatmap were drew by R language (Mathsoft, Inc., Cambridge, MA, USA) on the platform of Illumina MiSeq sequencing.

Matching and quantification of the volatile components were performed using the National Institute of Standards and Technology database (NIST 05s). For MS, compounds were selected with a matching degree greater than 80%, combing with the retention time of C7–C40, and using the relative signal intensity of amyl acetate or sec-octanol to calculate the area percentage of each peak.

Redundancy analysis (RDA) was performed using Canoco 5 software (Cabit Information Technology Co., Ltd., Shanghai, China) and heatmap analysis was performed using Heml software (https://cloud.oebiotech.com (accessed on 6 December 2023)). Cytoscape software (version 3.5.1, http://www.cytoscape.org/ (accessed on 6 December 2023)) was used to analyze the correlation between the microbial flora and volatile components.

All the data were performed in triplicate and displayed with mean value and standard deviation. Duncan test has been employed to evaluate the statistical significance of the determinations via variance analysis by comparative averages (one-way ANOVA) applying the SPSS v 21.0 software for Windows (IBM).

## 3. Results

### 3.1. Analysis of Physicochemical Indexes of FG and PM

As presented in Table 1, it becomes evident that the moisture content and acidity levels within FG samples procured from the base of the fermentation cellar (CB) emerged as the most pronounced among the three analyzed samples. This is congruent with the higher moisture and acidity levels observed within FG samples harvested from the base of the fermentation cellar in contrast to those discerned within the remaining samples. Furthermore, the FG obtained from CB showcases the lowest levels of reducing sugars and starch among the three samples, as delineated in Table 1. In consonance with this, Table 2 presents a comprehensive overview of the physicochemical metrics characterizing PM, encompassing attributes such as moisture content, pH levels, and ammonium concentrations.

### 3.2. Plate Counting of Culturable Microorganisms in FG and PM

As indicated in Table 3, discernible trends emerge within the comparative analysis of the four sample categories. The comprehensive colony count, coupled with yeast and mold levels, exhibited the highest values within CA. In contrast, the bacterial count within CA registered the lowest measurement at 5.74 ± 0.32 ^b^ log CFU/g. Transitioning to the mid-layer of FG (CM), a pattern emerges wherein bacterial levels outstrip mold and yeast counts. This identical trend manifests within FG samples obtained from the lower stratum (CB). The PM samples, in turn, showcased the most elevated bacterial levels, quantified at 2.90 ± 0.00 ^c^ log CFU/g. In striking juxtaposition, yeast levels within PM were noted as the most minimal, measuring at 2.95 ± 0.05 ^b^ log CFU/g.

### 3.3. Analysis of Microbial Community Structure in FG and PM

**Figure 1:**
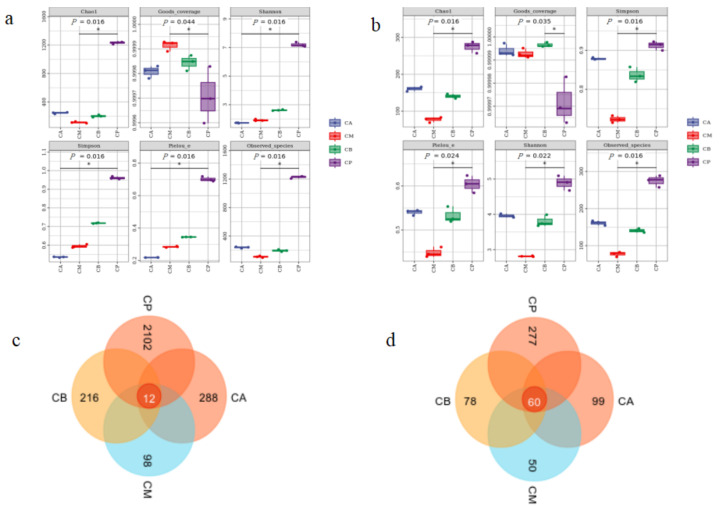
Microbial alpha diversity of fermented grains (FG) and pit-mud (PM): (**a**) alpha diversity of bacteria, (**b**) alpha diversity of fungi, (**c**) Venn diagram of microbial operational taxonomic unit (OTU) of bacteria, (**d**) Venn diagram of microbial OTU of fungi. For (**a**) and (**b**), the "*" indicates significant differences between groups.

Following meticulous quality control filtration, the FG samples (CA, CM, and CB) and PM (CP) yielded bacterial sequences numbering 124, 138–168, and 166, respectively. These sequences exhibited an average length spanning from 137 to 338 base pairs. Emanating from this, a cumulative total of 2912 bacterial operational taxonomic units (OTUs) were engendered via cluster analysis. Simultaneously, the fungal sequences numbered between 111,765 and 128,262, characterized by an average length ranging from 195 to 434 base pairs. Notably, this culminated in the generation of 698 fungal OTUs through cluster analysis. The extensive coverage of samples within each grouping surpassed the threshold of 0.999, complemented by a calculated *p* value of 0.016 for the Chao1 index (Figure 1a,b). Robust validations from the dilution curve, Shannon curve, and alpha diversity collectively affirm the representative nature of the acquired sequencing data of the samples (Figure 1 and Figure S2). Furthermore, the focal point of our analysis honed in on the predominant microbial species found within FG and PM, facilitated through the utilization of a clone library approach.

According to the application of the Shannon and Simpson indices, the portrayal of species number, evenness, and diversity across the samples was accomplished in previous studies. As depicted in Figure 1a,b, the Chao1 index for both bacterial and fungal entities within PM stood as the most elevated, thus elucidating that the total count of bacterial and fungal species within PM surpassed that of all other samples. Additionally, the Shannon and Simpson indices for both bacteria and fungi equally scaled the pinnacle within PM, further underscoring that PM exhibited the utmost uniformity and diversity among its bacterial and fungal species.

Within distinct groupings, a total of 12 bacterial operational taxonomic units (OTUs) manifested, with PM reigning supreme, amassing a noteworthy 2102 bacterial OTUs, thereby representing a substantial 72.18% of the cumulative bacterial OTUs present across the groups. This observation lends credence to PM as the epicenter of peak bacterial diversity (Figure 1c). On a similar note, the fungal realm exhibited the presence of 60 fungal OTUs dispersed among diverse groups, with PM again displaying its preeminence by harboring 277 fungal OTUs, encompassing 39.68% of the entire fungal OTU spectrum. This underscores the robust fungal diversity housed within PM (Figure 1d). Furthermore, the count of fungal OTUs within CA eclipsed those observed in CB and CM, effectively indicating that the spectrum of fungal diversity within CA stood as the most prolific among the diverse FG samples. These observations harmoniously align with the outcomes derived from the Shannon and Simpson indices.

### 3.4. Microbial Community Structure, Heatmap, and LEfSe Analysis of FG and PM

**Figure 2:**
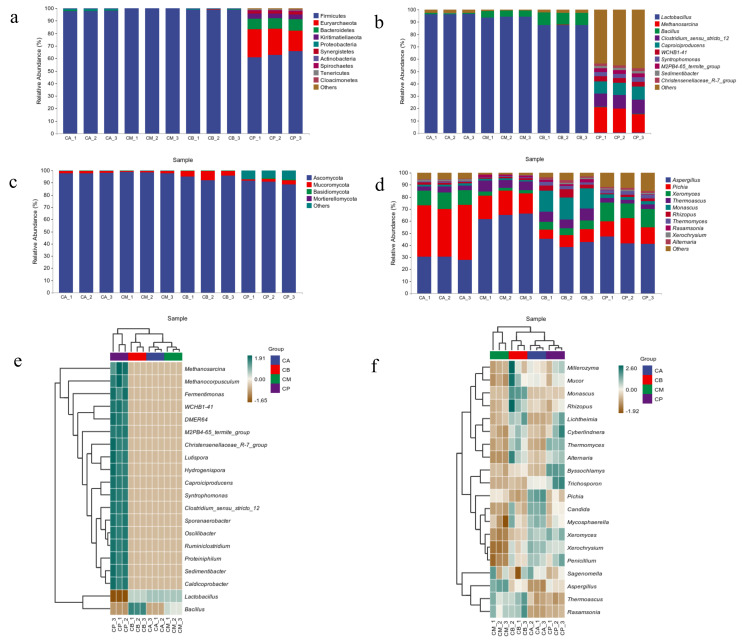
The microbial community structure and Heatmap analysis of fermented grains (FG) and pit-mud (PM): (**a**) bacteria at phylum level, (**b**) bacteria at genus level, (**c**) fungi at phylum level, (**d**) fungi at genus level, (**e**) heatmap of bacteria, (**f**) heatmap of fungi.

For bacteria, the dominant phylum was Firmicutes, and its relative abundance in different FG samples was greater than 90.00% (Figure 2a). However, the dominant phyla were Firmicutes and Euryarchaeota in PM. The bacteria in FG belong to two main genera: *Lactobacillus* and *Bacillus* (Figure 2b). The relative abundance of *Lactobacillus* in different FG samples was higher than 87.00%, therefore, *Lactobacillus* was the dominant bacterium in FG. For *Bacillus*, the relative abundance was higher than 9.80% in CB, which was higher than that in CA and CM.

The dominant genera in PM were *Methanosarcina* and *Clostridium_sensu_stricto_12*, which were different from those in FG, and their relative abundances were higher than 10.00% (Figure 2e). There were two bacterial genera with high abundance (more than 1.00%) at least, which were the important bacterial genera in FG and PM. In addition, there was no common dominance of bacterial genera in FG and PM.

As shown in Figure 2c, Ascomycota was the dominant fungal phylum in FG and PM, and their relative abundances in different samples were higher than 88.00%. The fungi in the FG can be classified into six main genera: *Aspergillus*, *Pichia*, *Xeromyces*, *Thermoascus*, *Monascus*, and *Rhizopus (*Figure 2d). In different FG samples, the relative abundance of *Pichia* was higher than 9.00%, and its relative abundance in CM or CB was lower than that in CA. We noticed that the relative abundance of *Thermomyces* in PM was 2.92%, which was similar to that in CB (2.45%) and higher than those in CA (0.71%) and CM (0.66%), indicating that the microbial community in CB was greatly affected by that in PM.

According to Figure 2f, at least six fungal genera with high abundances (more than 1.00%) were considered to be the important genera. For the bottom of FG (CB), the relative abundances of *Pichia*, *Monascus*, and *Aspergillus* were 9.31%, 17.49%, and 4.21%, respectively, indicating that they were dominated by esterification microorganisms.

**Figure 3:**
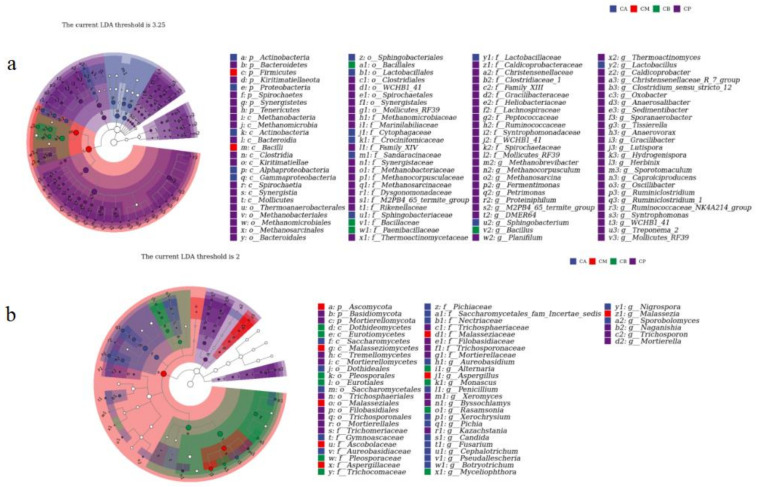
LEfSe analysis of fermented grains (FG) and pit-mud (PM): (**a**) LEfSe analysis of differential species annotated clade of bacteria, (**b**) LEfSe analysis of differential species annotated clade of fungi.

There were 325 bacteria upregulated in PM, including those in Clostridiales and Lactobacillales family, and *Weissella* genera; 33 fungi were upregulated, including those in Aspergillaceae and Mucoraceae family, and *Candida* genera (Figure 3a,b). Green and red nodes indicate that these taxa exhibited significant intergroup differences and were more abundant in different samples represented by color. Meanwhile, letters indicate the names of taxa that differ significantly between different groups. Herein, Firmicutes phylum and *Bacillus* genera, and Ascomycota phylum and Aspergillaceae family differed significantly between samples groups.

### 3.5. Principal Component Analysis (PCA), Hierarchical Clustering Analysis, and Network Correlation Analysis of Microbial Community in FG and PM

**Figure 4:**
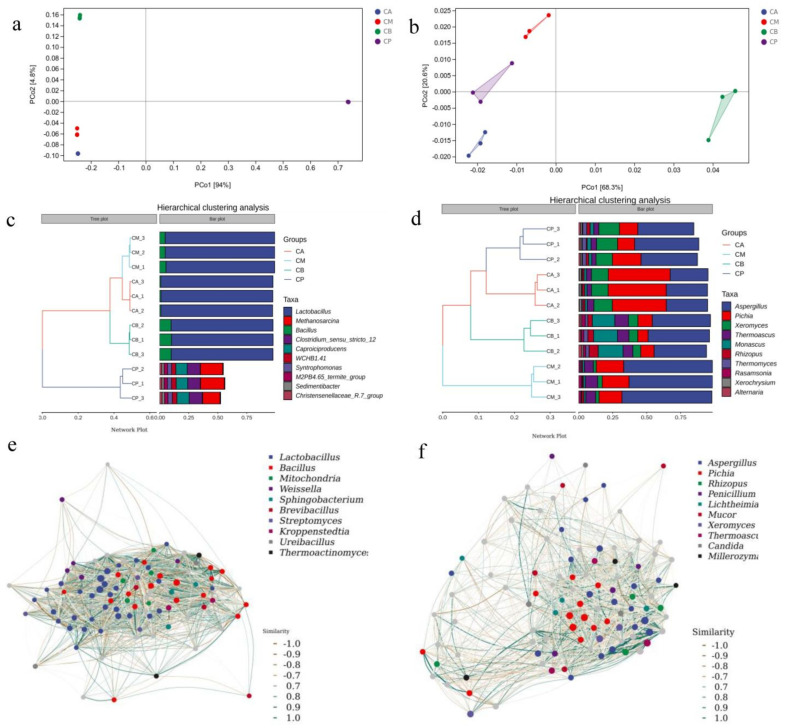
Principal component analysis (PCA), hierarchical clustering analysis and network correlation analysis of bacteria and fungi at the genus level: (**a**) PCA of bacteria, (**b**) PCA of fungi, (**c**) Hierarchical clustering of bacteria, (**d**) Hierarchical clustering of fungi, (**e**) Network correlation analysis of bacteria, and (**f**) Network correlation analysis of fungi.

Regarding bacteria (Figure 4a), sample variations yielded figures of 94.00% (PCo1) and 4.80% (PCo2), showcasing a pronounced regional demarcation. Notably, the CA, CM, CB, and PM samples displayed similarities, indicating a consistent and stable bacterial composition across the sample cohorts. Conversely, samples sourced from FG (CA, CM, and CB) and PM exhibited a scattered distribution, underscoring discernible dissimilarities in bacterial diversity between PM and FG. Shifting focus to fungi (Figure 4b), the highest variation values materialized as 68.30% (PCo1) and 20.60% (PCo2), leading to a dispersion of samples. This pattern substantiates discernible distinctions in fungal diversity between FG and PM.

In the hierarchical clustering diagram shown in Figure 4c,d, the FG samples (CA, CM, and CB) were similar in bacterial diversity, and the three bacterial genera with high relative abundances were *Lactobacillus*, *Methanosarcina*, and *Bacillus*. Similarly, the PM, CA, and CB samples had similar fungal diversity, and the three fungal genera with high relative abundances were *Aspergillus*, *Pichia*, and *Xeromyces*.

To clarify the assembly differences of community species and identify key bacterial species that can leverage changes in community composition, the microbial association networks were used. *Bacillus*, *Lactobacillus*, and *Weissella* showed a close positive correlation with other bacterial genera (Figure 4e), indicating *Bacillus* and *Lactobacillus* had pronounced negative correlations. Meanwhile, *Bacillus* was negatively correlated with *Ureibacillus* and *Thermoactionmyces*. In addition, *Aspergillus*, *Pichia*, and *Rhizopus* were positively correlated with other fungal genera, a pronounced negative correlation between *Aspergillus*, *Candida*, and *Millerozyma* (Figure 4f). In summary, these three genera were the main fungal microflora in the FG of the different layers and in the PM from the bottom of the fermentation cellar.

### 3.6. Analysis of Volatile Components in FG and PM

**Figure 5:**
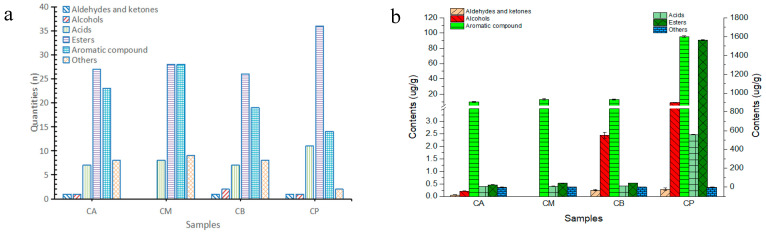

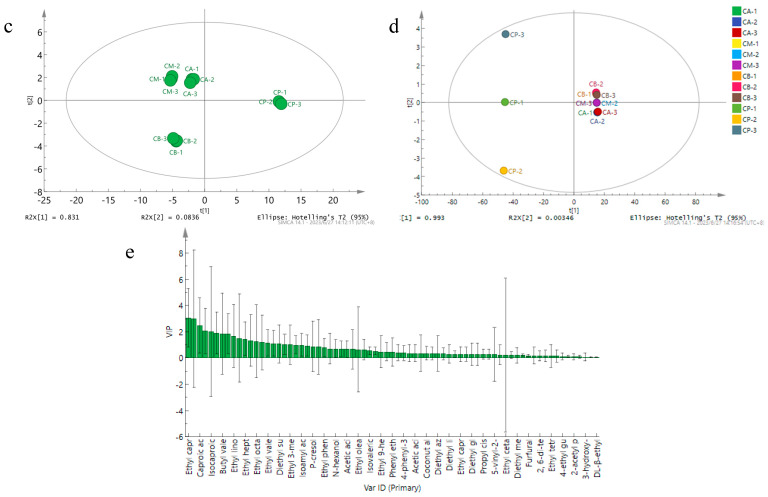
The categories and contents of volatile components in fermented grains (FG) and pit-mud (PM): (**a**) quantity of volatile components; (**b**) content of volatile components; (**c**) principal component analysis (PCA) of volatile components; (**d**) partial least squares discriminant analysis (PLS-DA); (**e**) Variable importance of projective (VIP).

A total of 73 volatile components were detected within FG (including the internal standard). Among these, 14 substances took center stage as the principal volatile components, encompassing ethyl hexanoate, ethyl octanoate, and caproic acid (Tables S1 and S2). In tandem, PM exhibited the presence of 65 volatile components, including caproic acid, acetic acid, and ethyl caproate (Tables S1 and S2). Significantly, the concentrations of acids and esters within PM surpassed those evident in the diverse FG samples. Among these, 26 volatile components were common to both FG and PM samples, whereas 54 volatile components were shared among distinct FG samples. However, it’s noteworthy that the CB and PM samples presented 32 shared volatile components, signifying the influence of FG components from the lower strata of the fermentation cellar on the volatile makeup of PM. The volume and composition of volatile components within PM emerged as the most prominent among the quartet of sample types, with CB boasting the highest alcohol content among the three FG samples (Figure 5a,b).

The cumulative contribution rate of corresponding features within the first two principal components reached 91.5%, as depicted in Figure 5c. This value aptly encapsulates a substantial portion of the pertinent flavor-related information. Moving to Figure 5d, the cumulative contribution rate of corresponding features in the first two principal components soared to 99.6%, affirming the model’s capability to accurately reflect a remarkable 99.6% alteration in the dataset. Furthermore, all samples resided comfortably within the 95% confidence interval of Hotelling’s T2, an affirmation of the model’s stability, dependability, and its capacity to adeptly distinguish between distinct volatile components across varied samples. The application of partial least squares discriminant analysis (PLS-DA) unveiled that FG samples clustered within specific regions (Figure 5d), a pattern divergent from that observed for PM samples. This contrast implies discernible dissimilarities between the volatile components present in each FG and PM sample.

VIP denotes the weight value ascribed to the variables within the PLSDA model. A higher VIP value correlates with a more substantial contribution magnitude. Typically, VIP values exceeding 1 signify pivotal differentiating components among samples, whereas VIP values below 1 exert negligible influence on sample differentiation. Illustrated in Figure 5e, a total of 33 compounds with VIP values surpassing 1 were identified through screening. Remarkably, caproic acid emerged with the highest VIP value, thereby designating it as the distinctive constituent accentuating disparities between samples.

### 3.7. RDA and Correlation Perfume Circos of Microbial Community with Volatile Components of FG

**Figure 6:**
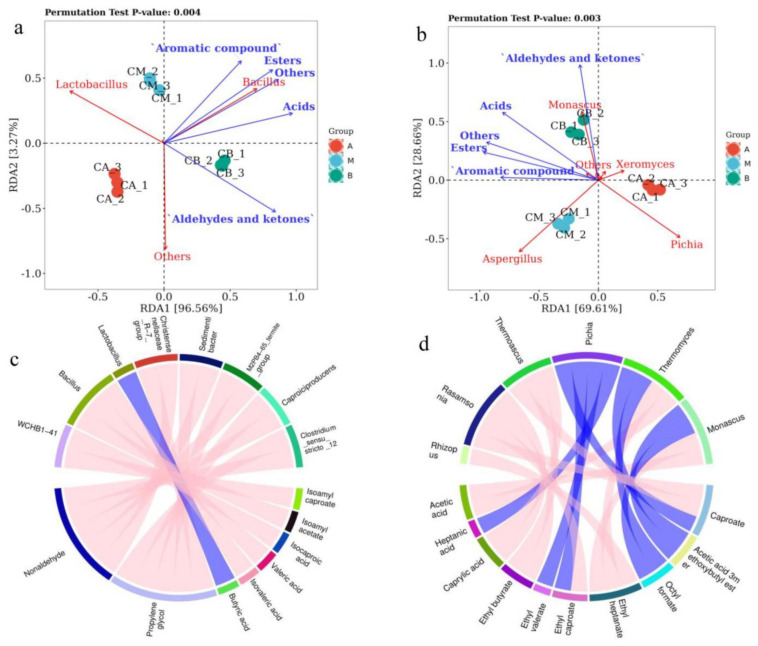
RDA analysis and correlation perfume Circos of dominant microorganisms at genus level in fermented grains (FG): (**a**) RDA analysis of volatile components and bacterial genera; (**b**) RDA analysis of volatile components and fungal genera; (**c**) correlation of perfume Circos of bacteria with volatile components; (**d**) correlation of perfume Circos of fungi with volatile components.

In the realm of groups, the top two axes of Redundancy Analysis (RDA) comprehensively accounted for 96.56% and 69.61% of the variance in metabolites. This robust explanation underscores the potent correlations between microbial communities and volatile components within FG. As visually depicted in Figure 6a, a notable correlation surfaced between the bacterial genera *Bacillus* and *Lactobacillus* and the anabolic processes of a majority of volatile components. Likewise, within the fungal domain, the genera *Aspergillus*, *Pichia*, and *Monascus* exhibited pronounced correlations with the anabolic pathways of a significant array of volatile components (Figure 6b).

Figure 6c illustrates a positive correlation between *Lactobacillus* and the anabolism of butyric acid, while Bacillus exhibited a negative correlation with the anabolic processes of isovaleric acid, valeric acid, and isocaproic acid. Figure 6d presents *Pichia* as the fungal genus boasting the most substantial number of connections, with a positive correlation established between *Pichia* and the anabolic processes of heptanic acid, ethyl valerate, ethyl caproate, and caproate. *Monascus*, on the other hand, positively correlated with octyl formate anabolism, but conversely displayed a negative correlation with the anabolic processes of ethyl hentanate and acetic acid. Evidently, *Lactobacillus*, *Bacillus*, *Aspergillus*, *Pichia*, and *Monascus* stand as central genera of microbial, which are pivotal in shaping the metabolism of volatile components within FG. Nevertheless, further exploration is requisite to establish and delve into the functional correlations between the core microbiota and volatile components within the domain of Baijiu brewing.

## 4. Discussion

### 4.1. Physicochemical Indexes and Plate Counting of Culturable Microorganisms of FG and PM

This observation signifies a heightened utilization of reducing sugars and starch in CB when juxtaposed with the scenarios in CA and CM. The pH, humus content, and the flourishing of microbial flora within PM are intrinsically linked to its moisture levels. Physicochemical indices, encompassing parameters like moisture, acidity, reducing sugar, and starch, wield pivotal influence over the microbial community structure of FG. This influence is further compounded by microbial metabolic processes [17,23,24]. It is noteworthy that an optimal pH for PM serves to catalyze alcohol fermentation and facilitate the creation of precursor substances of aromas within baijiu [16]. The physicochemical metrics characterizing PM samples procured during our research with those encapsulating 20-year aged PM [16].

The outcomes of plate counting within this study have highlighted discernible differences in culturable microorganism counts between FG and PM. The comprehensive colony count, coupled with yeast and mold levels, exhibited the highest values within CA. During baijiu fermentation, saccharifying enzymes are mainly produced by moulds, such as *Rhizopus*, *Aspergillus*, and *Monascus*. Among the culturable fungi, *Paecilomyces* spp. had the highest glucoamylase activity, however *Aspergillus oryzae* showed the highest α-amylase activity. The reducing sugar content in the fermentation process were consistent with the growth of *Paecilomyces* spp. and *Aspergillus oryzae*. Therefore, both fungi play important roles in the amylase-mediated starch hydrolysis [16,17,25].

Within the intricate tapestry of baijiu fermentation, yeast community structure occupies a complex role and holds utmost importance. At the inception of baijiu production, the amalgamation of cooked grains with water, and occasionally supplementary ingredients, forms the mash. This pivotal stage sees naturally occurring yeasts on the grains or those introduced through starter cultures spearheading the fermentation process. These yeasts diligently transform the grain-based sugars into alcohol and carbon dioxide. Concurrently, they engender an array of flavor compounds, including alcohols, esters, and volatile constituents, all of which contribute to baijiu’s distinctive aroma and flavor profile [15]. The contribution of yeasts extends deeply into shaping the overarching flavor of baijiu. Unique strains of yeast initiate diverse flavor compounds during fermentation, ushering in an array of sensory characteristics spanning from fruity and floral to even earthy undertones.

The meticulous selection and management of yeasts wield the power to substantially influence the final aromatic and flavor notes of the distilled spirit [3]. In the present study, our focal point shifted towards the spatial distribution of yeast across varied locations within the cellar. Notably, Wang et al. (2019) illuminated the discrepancy in yeast density between upper- and lower-layers during baijiu fermentation [25]. Evidently, yeast populations within different strata give rise to diverse aromatic constituents. Our findings seamlessly align with the conclusions of Wang et al. [25].

### 4.2. Microbial Community Structure of FG and PM

The portrayal of species number, evenness, and diversity across different samples were accomplished according to the application of the Shannon and Simpson indices, a methodology well-versed in previous studies [10,26]. Beyond the purview of microbial community analysis, it was discerned that the bacterial genera residing within FG diverged from those inhabiting PM. In contrast, the fungal genera identified in PM exhibited congruence with those present in FG, a congruity that concurs with previous research findings [7,8]. *Lactobacillus* is an important lactic acid-producing bacterium in FG, and Lactic acid is an important flavoring substance in baijiu [10]. *Bacillus* produces fragrances, including pyrazines, acids, methyl esters, and other flavor substances [16]. Notably, *Lactobacillus*, a prominent lactic acid-producing bacterium within FG, significantly contributes to the production of lactic acid, a pivotal flavor compound in baijiu [10,27]. Conversely, Bacillus generates metabolites such as pyrazines, acids, and methyl esters, constituting integral flavor components [28]. Therefore, the quality of baijiu distilled from CB was better than that of baijiu distilled from CA and CM [5]. As a major phylum, *Ascomycota* plays an important role in brewing different aroma styles of baijiu, such as Luzhou-flavored and soy sauce-flavored baijiu [28].

Baijiu fermentation represents an intricate microbial interplay involving yeasts, bacteria, and molds, characterized by a dynamic succession process. The microbial diversity during baijiu fermentation undergoes a series of discernible transformations over time, collectively bestowing the ultimate spirit with its distinct aroma and flavor profile [29,30]. In the journey of fermentation, microorganisms continuously undergo domestication, subject to the influences of environmental factors and intricate microbial interactions. These dynamics lead to variations in the composition and abundance of bacterial and fungal species within FG and PM [31]. Evidently, the microbial community present in the CB, sourced from the bottom strata of FG, bears significant influence from the PM milieu. Temporally, the domination of specific microorganisms can experience shifts. As the fermentation process unfolds, the equilibrium between various yeast and bacterial species evolves in tandem with changing conditions, thereby establishing a dynamic microbial ecosystem within the fermentation matrix [11,32,33].

### 4.3. Relationships between Microbial Communities and Volatile Compounds

In tandem with the volatile components, FG is directly subjected to distillation to yield raw baijiu. During the course of FG fermentation, any perturbation in the process variables can initiate cascading effects, impacting microbial communities and metabolites. In addition, the presence of yellow pulp water, engendered through the degradation of starch and other macromolecules during brewing process, is replete with constituents such as lactic acid, ethanol, and acetic acid [11]. In addition, PM stands as an intricate repository of diverse and intricate metabolites, encompassing an array of flavor components that profoundly influence the flavor profile and overall caliber of baijiu [34]. Throughout baijiu fermentation, microorganisms undergo migration, engendering flavor shifts through their metabolic actions [35,36]. Microorganisms, encompassing yeasts and bacteria, find their way into the fermentation matrix via the raw materials or the fermentation environment [9]. Their introduction triggers colonization and rapid proliferation within the mixture. Yeasts hold a pivotal role in the initial fermentation phase, metabolizing sugars present in the raw materials (e.g., grains) to generate alcohol and carbon dioxide. Distinct yeast strains yield diverse flavor compounds such as esters and higher alcohols, shaping the nascent aroma and taste of baijiu [11,29,37,38]. Concurrently, as yeasts and bacteria thrive, a diverse array of metabolic byproducts emerges. These encompass volatile substances like esters, aldehydes, and organic acids, orchestrating the sensory attributes of baijiu [29].

The multiplicity of microorganisms coupled with their metabolic prowess lends intricacy to the flavor spectrum. With fermentation’s progression, microorganisms proliferate and partake in mutual interactions. Some microorganisms engender enzymes that dismantle intricate compounds in the raw materials, liberating additional flavor precursors [38]. These compounds then undergo further transformations into intricate flavors over the course of fermentation. The latter stages are marked by heightened bacterial activity, particularly that of lactic acid bacteria. These microorganisms contribute to the conversion of malic acid to lactic acid through malolactic fermentation [39,40,41]. These organic acids are considered precursors for the generation of esters, serving as pivotal flavor constituents within baijiu [42]. This metabolic event reduces acidity while introducing nuances reminiscent of creaminess or butteriness to baijiu. The interplay between diverse microorganisms and their metabolic endeavors orchestrates an evolving tapestry of flavors and aromas throughout fermentation [43,44,45].

Temporally, the equilibrium of microbial populations and their contributions undergoes shifts, culminating in a dynamic flavor profile. In baijiu fermentation, the amalgamation of grain mixture with PM triggers a crossroads of microorganisms, enabling the migration from PM to engender an array of flavor compounds, prominently including alcohols, fatty acids, and esters [46,47]. In the present study, CB samples entailed FG sedimentation within the lower strata of the pits, interfacing with PM. Consequently, the microbial community and volatile compounds within CB were notably influenced by PM, necessitating further exploration of these correlations.

As we know, *Pichia* plays a critical role in aroma metabolism, whereas *Monascus* plays a vital role in promoting the generation of esters. Meanwhile, *Aspergillus* can produce a variety of enzymes, organic acids and fatty acids, which are facilitate to the generation of aromatic esters [4,27,45,48]. Given the robust correlations between microbial communities and volatile components within FG, this study meticulously examined and interrelated culturable microorganisms, microbial communities, and volatile components within FG. *Monascus*, an exceptional functional fungus present within FG, assumes a pivotal role in enhancing ester formation throughout baijiu fermentation [4].

Our investigation unveiled *Lactobacillus, Bacillus, Aspergillus, Pichia,* and *Monascus* as the fundamental microbial genera within FG. Notably, *Lactobacillus* has been documented to contribute to the metabolism of lactic acid, ethanol, and acetic acid, making it a cornerstone functional microorganism responsible for augmenting acidity [49]. *Pichia*, prominent among the non-alcoholic yeasts in FG, assumes a key role in generating volatile compounds during baijiu fermentation [50]. *Monascus*, meanwhile, emerges as a pivotal contributor in promoting the synthesis of esters, such as ethyl acetate and ethyl caproate [27]. In summary, the majority of volatile components in FG, spanning alcohols, acids, and esters, displayed notable correlations with the core microbial genera of *Lactobacillus, Bacillus, Aspergillus, Pichia*, and *Monascus* instrumental in the production and metabolism of volatile components within FG.

## 5. Conclusions

In summary, this study meticulously examined and interrelated the physicochemical indices, culturable microorganisms, microbial communities, and volatile components within FG and PM. The outcomes revealed that *Aspergillus*, *Pichia*, and *Rhizopus* stood out as the predominant fungal microflora in FG from various layers and PM from the bottom of the fermentation cellar. Significantly, the interplay between microbial communities and volatile compounds within FG, particularly those from lower strata, exhibited substantial influence from the counterparts in PM. Notably, the CB and PM samples shared 32 common volatile components, with the PM samples displaying elevated levels of acids and esters compared to their FG counterparts. Through the lens of Redundancy Analysis (RDA), it was evident that a robust correlation linked the majority of volatile components in FG encompassing alcohols, acids, and esters to *Lactobacillus*, *Bacillus*, *Aspergillus*, *Pichia*, and *Monascus*. These core microbial genera play pivotal roles in the metabolization of volatile components within FG. This investigation renders valuable insights into the intricate interplay of microbial composition and volatile components between FG and PM, fostering potential applications in deciphering fermentation mechanisms, refining brewing techniques, and elevating the flavor profile of CFB.

## Figures and Tables

**Table 1:** Physicochemical indexes of FG.

Samples	Moisture Content (%)	Acidity (n mol/10 g)	Starch Content (g/100 g)	Reducing Sugars Content (g/100 g)
CA	62.05 ± 0.21 ^b^	4.05 ± 0.07 ^b^	8.58 ± 2.34 ^a^	0.43 ± 0.32
CM	63.68 ± 0.18 ^b^	4.40 ± 0.11 ^b^	7.63 ± 1.05 ^a^	0.35 ± 0.15
CB	67.67 ± 0.27 ^a^	5.39 ± 0.05 ^a^	5.75 ± 1.63 ^b^	0.29 ± 0.11

The abbreviations “CA,” “CM”, and “CB” indicate the fermented grains obtained from the upper, middle, and bottom layers, respectively, of the fermentation cellar. Values are given as mean ± standard deviation of three biological replicates. Letters indicate the level of significant difference (*p* < 0.05) as determined with one-way ANOVA analysis.

**Table 2:** Physicochemical indexes of PM.

Sample	Moisture (%)	pH	Ammonium (mg/100 g)	Available-P (mg/kg)	Available-K (mg/kg)	Calcium (g/kg)	Humus (g/kg)
CP	46.58 ± 0.02	5.53 ± 0.31	31.48 ± 0.15	128.36 ± 0.01	5211.36 ± 30.01	0.86 ± 0.08	219.36 ± 0.01

“CP” indicates the pit mud sample collected from the bottom of the fermentation cellar. Values are given as mean ± standard deviation of three biological replicates.

**Table 3:** Total number of culturable microorganisms determined by plate counting method in PM and FG.

Samples	Total Colony Count (PCA, log CFU/g)	The Total Number of Bacterial Colonies (LB, log CFU/g)	The Total Number of Mold Colonies (CD, log CFU/g)	The Total Number of Yeast Colonies (YPD, log CFU/g)
CA	7.55 ± 0.04 ^a^	5.74 ± 0.32 ^b^	7.54 ± 0.33 ^a^	6.37 ± 0.03 ^a^
CM	7.31 ± 0.64 ^a^	7.73 ± 0.16 ^a^	7.37 ± 3.33 ^a^	0.26 ± 1.58 ^c^
CB	7.21 ± 1.20 ^a^	7.64 ± 0.09 ^a^	6.38 ± 4.34 ^ab^	4.46 ± 1.69 ^ab^
CP	2.57 ± 0.13 ^b^	2.90 ± 0.00 ^c^	2.08 ± 1.90 ^b^	2.95 ± 0.05 ^b^

The abbreviations “CA,” “CM”, and “CB” indicate the fermented grains obtained from the upper, middle, and bottom layers, respectively, of the fermentation cellar. “CP” indicates the pit mud sample collected from the bottom of the fermentation cellar. Values are given as mean ± standard deviation of three biological replicates. Letters indicate the level of significant difference (*p* < 0.05) as determined with one-way ANOVA analysis.

## Data Availability

Data is contained within the article or Supplementary Material.

## Supplementary Materials

The following supporting information can be downloaded at: https://www.mdpi.com/article/10.3390/foods13020203/s1, Figure S1. Fermentation cellar for compound-flavor baijiu (CFB) and sketch map of sampling during ultra-long fermentation. (a) Fermentation cellars; (b) Sketch map of the sampling; Figure S2. Rarefaction, Shannon curves and Species accumulation curves of bacterial and fungal sequences and species from fermented grain and pit mud samples. (a) Rarefaction curves of bacterial sequences, (b) Shannon curves of bacterial species, (c) rarefaction curves of fungal sequences, (d) Shannon curves of fungal species, (e) species accumulation curve of bacteria, and (f) species accumulation curve of fungi; Table S1. Categories and contents of volatile components in Pit-mud and fermented grains of different depths in fermentation cellars of Compound-flavor Baijiu; Table S2. Numbers of volatile components in Pit-mud and fermented grains of different depths in fermentation cellars of Compound-flavor Baijiu.
