# Supplementary material for: Comparison of the Correlations of Microbial Community and Volatile Compounds between Pit-Mud and Fermented Grains of Compound-Flavor Baijiu
Source: Foods. 2024 Jan 8;13(2):203. doi: 10.3390/foods13020203 (PMC10814010; doi:10.3390/foods13020203)
Supplement: Supplementary file 1 [file foods-13-00203-s001.zip › foods-2787640-supplementary.pdf]

Supplementary Material

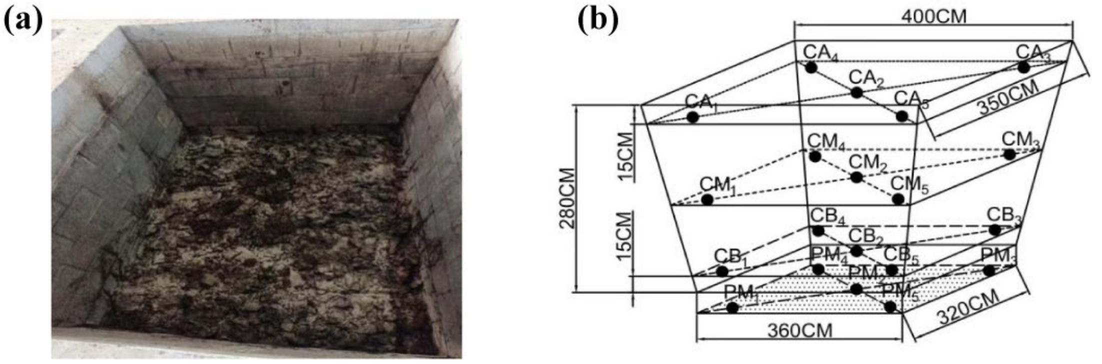

Figure S1. Fermentation cellar for compound flavor baijiu and sketch map of sampling during ultra-long fermentation. a) Fermentation cells; b) Sketch map of the sampling.

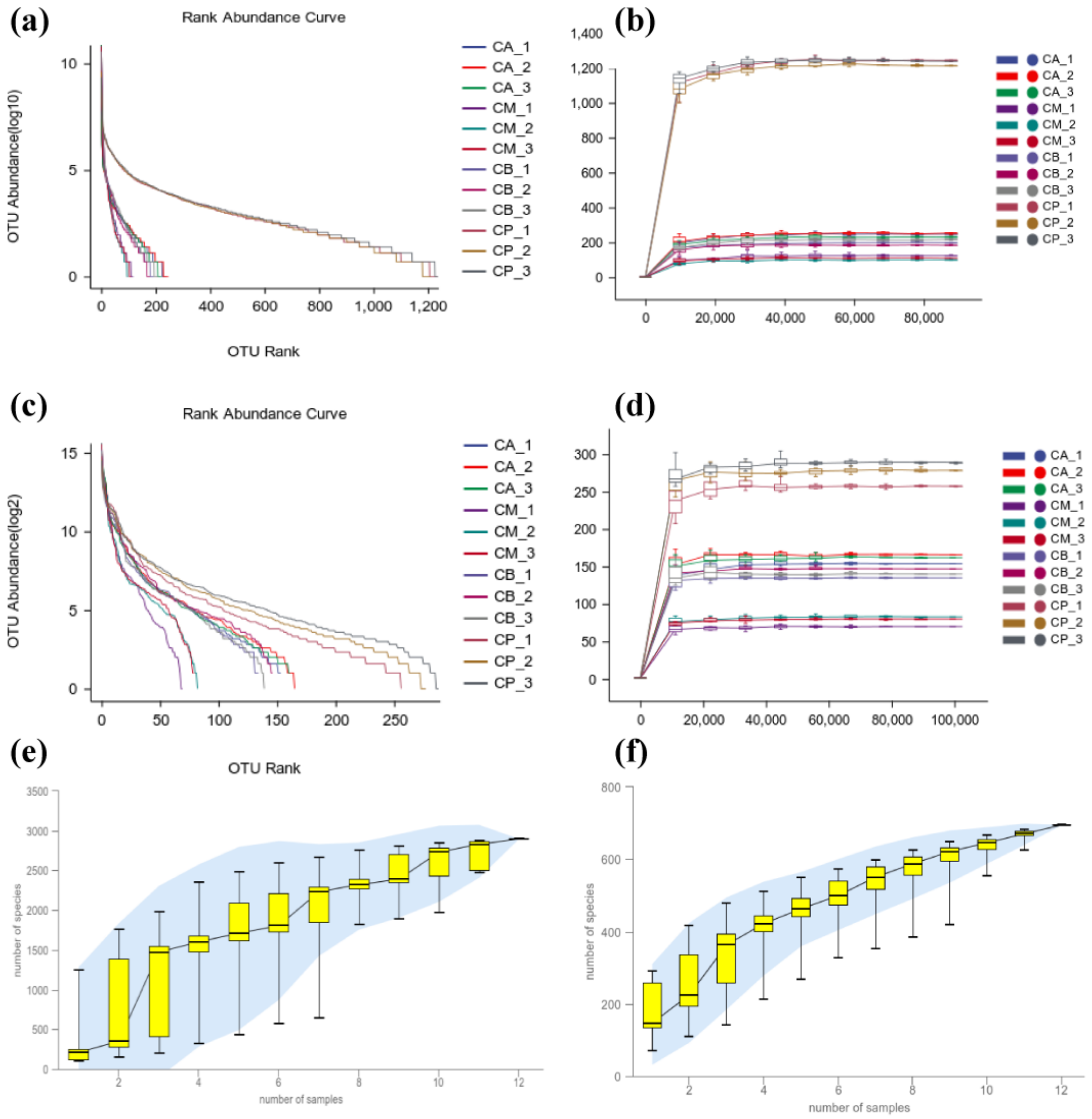

Figure S2. Rarefaction, Shannon curves, and species accumulation curves of bacterial and fungal sequences and species from fermented grain and pit mud samples. a) Rarefaction curves of bacterial sequences, b) Shannon curves of bacterial species, c) rarefaction curves of fungal sequences, d) Shannon curves of fungal species, e) species accumulation curve of bacteria, and f) species accumulation curve of fungi.

Table S1. Categories and contents of volatile components in pit mud and fermented grains of different depths in fermentation cellars of compound-flavor baijiu.

Kinds	Volatile components	Contents of volatile components (μg/g)			
		Different samples of fermented grains			Pit-mud
		CA	CM	CB	CP
Aldehydes and ketones	Nonaldehyde	0.06 ± 0.00	ND	ND	ND
	2-Cyclohexen-1-one, 2,4,4-trimethyl-3-(3-methylbutyl)-	ND	ND	0.25 ± 0.05	ND
	E-15-heptadecenic aldehyde	ND	ND	ND	0.29 ± 0.04
Alcohols	N-hexanol	ND	ND	2.35 ± 0.30	8.94 ± 0.26
	Propylene glycol	0.21 ± 0.02	ND	ND	ND
	1-nonyl alcohol	ND	ND	0.09 ± 0.01	ND
Acids	Acetic acid	1.03 ± 0.16	0.82 ± 0.10	1.13 ± 0.15	27.26 ± 2.29
	Butyric acid	0.86 ± 0.03	1.40 ± 0.05	2.83 ± 0.45	ND
	Isobutyric acid	ND	ND	ND	5.43 ± 0.58
	Isovaleric acid	0.61 ± 0.11	0.84 ± 0.02	1.20 ± 0.07	8.56 ± 2.45
	Valeric acid	0.57 ± 0.03	0.97 ± 0.02	1.47 ± 0.07	50.38 ± 11.11
	Isocaproic acid	ND	0.07 ± 0.00	ND	1.89 ± 0.01
	Caproic acid	0.29 ± 0.07	0.68 ± 0.05	0.71 ± 0.08	338.44 ± 37.16
	Heptanic acid	0.29 ± 0.01	0.46 ± 0.04	0.54 ± 0.01	70.68 ± 0.86
	Caprylic acid	1.08 ± 0.05	2.50 ± 0.05	2.80 ± 0.21	52.35 ± 0.33
	Nonaic acid	ND	ND	ND	1.66 ± 0.24
	N-capric acid	ND	ND	ND	0.73 ± 0.14
Esters	Ethyl butyrate	1.08 ± 0.05	2.50 ± 0.05	2.80 ± 0.21	59.08 ± 2.07
	Propyl butyrate	ND	ND	ND	6.27 ± 0.98
	Butyl butyrate	ND	ND	ND	32.10 ± 3.10
	Isoamyl butyrate	ND	ND	ND	4.38 ± 0.09
	Isoamyl acetate	0.20 ± 0.05	0.26 ± 0.02	0.32 ± 0.02	ND
	Hexyl acetate	ND	ND	ND	1.86 ± 0.27
	Octyl 3-methyl butyrate	ND	ND	ND	1.63 ± 0.07
	Ethyl valerate	1.39 ± 0.00	2.31 ± 0.17	2.72 ± 0.06	76.33 ± 1.72
	Isoamyl isovalerate	ND	ND	ND	0.96 ± 0.21
	Isoamyl n-valerate	ND	ND	ND	1.67 ± 0.11
	Octyl acetate	ND	ND	ND	0.64 ± 0.10

Propyl caprylate	ND	ND	ND	8.27 ± 0.87
Octyl caprylate	ND	ND	ND	0.81 ± 0.28
Ethyl nonanoate	ND	ND	ND	13.49 ± 0.66
Ethyl caproate	6.64 ± 0.37	16.36 ± 0.21	20.57 ± 1.15	507.58 ± 14.74
Octyl caproate	ND	ND	ND	7.36 ± 0.83
Ethyl 5-methylcaproate	ND	ND	ND	0.51 ± 0.07
Ethyl heptanate	0.06 ± 0.00	ND	0.49 ± 0.04	119.73 ± 13.85
Heptyl heptylate	ND	ND	ND	31.29 ± 1.44
Propyl caprate	ND	ND	ND	0.08 ± 0.05
Heptanoic acid,3-methylbutyl ester	ND	ND	ND	2.29 ± 0.19
Butyl caproate	0.40 ± 0.01	0.08 ± 0.01	0.17 ± 0.02	188.03 ± 3.92
Ethyl caprylate	0.40 ± 0.01	0.72 ± 0.03	0.55 ± 0.02	236.19 ± 25.99
Octanoic acid, 3-methylbutyl ester	ND	ND	ND	1.45 ± 0.16
Isoamyl caproate	ND	0.31 ± 0.01	ND	26.35 ± 2.26
Ethyl 2-hydroxy-4-methylvalerate	2.37 ± 0.01	2.59 ± 0.07	2.80 ± 0.13	ND
Octyl formate	1.16 ± 0.01	1.20 ± 0.01	1.00 ± 0.15	ND
Acetic acid 3-methoxybutyl ester	1.16 ± 0.01	1.20 ± 0.01	1.00 ± 0.15	ND
Caproate	ND	0.14 ± 0.01	0.18 ± 0.01	200.97 ± 1.82
Propyl caproate	ND	ND	ND	20.99 ± 2.94
Propyl caproate	ND	ND	ND	0.32 ± 0.01
Amyl caproate	ND	ND	ND	1.87 ± 0.27
Butyl valerate	1.39 ± 0.00	2.31 ± 0.17	2.72 ± 0.06	ND
Ethyl levulinate	0.11 ± 0.01	0.15 ± 0.01	0.12 ± 0.02	4.72 ± 0.79
Ethyl caprate	0.27 ± 0.01	0.30 ± 0.00	0.21 ± 0.01	ND
Diethyl methyl succinate	0.03 ± 0.01	0.07 ± 0.00	ND	ND
Diethyl succinate	2.33 ± 0.06	2.75 ± 0.03	3.11 ± 0.47	ND
Diethyl glutarate	0.12 ± 0.01	0.20 ± 0.00	0.18 ± 0.09	ND
Ethyl 3-hydroxycaprylate	0.06 ± 0.01	0.12 ± 0.02	0.10 ± 0.03	ND
Isoamyl succinate	0.05 ± 0.00	0.08 ± 0.02	0.07 ± 0.03	ND
Ethyl undecanoate	ND	ND	ND	0.42 ± 0.01
Ethyl tetradecanoate	0.19 ± 0.01	0.26 ± 0.05	0.17 ± 0.02	ND
Propyl cis-9-tetradecenoate	0.01 ± 0.01	0.02 ± 0.00	ND	ND
Diethyl linoleate	0.05 ± 0.01	0.06 ± 0.01	0.02 ± 0.01	ND
Ethyl pentadecanoate	0.05 ± 0.00	0.07 ± 0.02	0.05 ± 0.01	0.50 ± 0.02
Diethyl azelaic acid	0.16 ± 0.04	0.25 ± 0.00	0.12 ± 0.02	ND

	Ethyl cetanoate	2.16 ± 0.11	3.57 ± 0.42	2.27 ± 0.20	4.56 ± 0.06
	Ethyl 9-hexadecenate	0.18 ± 0.02	0.28 ± 0.06	0.10 ± 0.01	0.04 ± 0.00
	Ethyl octadecanoate	0.47 ± 0.05	0.88 ± 0.18	0.03 ± 0.02	ND
	Ethyl oleate	0.18 ± 0.02	0.77 ± 0.08	0.47 ± 0.03	0.72 ± 0.08
	Ethyl linoleate	0.77 ± 0.08	0.47 ± 0.05	0.19 ± 0.02	0.18 ± 0.03
	3-hydroxy-4-methoxybenzaldehyde	0.17 ± 0.02	0.17 ± 0.00	0.24 ± 0.01	0.89 ± 0.02
	Naphthalene	0.06 ± 0.00	0.11 ± 0.01	0.09 ± 0.00	ND
	O-dimethyl ether	0.04 ± 0.00	0.06 ± 0.00	0.08 ± 0.02	ND
	Ethyl phenylacetate	1.11 ± 0.04	1.59 ± 0.05	1.70 ± 0.38	11.97 ± 1.28
	Phenyl ethyl acetate	1.17 ± 0.01	1.45 ± 0.05	1.40 ± 0.33	ND
	3, 4-dimethyl benzaldehyde	0.17 ± 0.05	0.46 ± 0.14	ND	ND
	Guaiacol	0.11 ± 0.01	0.15 ± 0.04	0.16 ± 0.03	ND
	Benzyl alcohol	0.19 ± 0.00	0.32 ± 0.01	0.38 ± 0.06	ND
	Ethyl phenylpropionate	0.45 ± 0.05	0.97 ± 0.08	1.06 ± 0.03	35.06 ± 6.76
	2, 6-di-tert-butylp-cresol	0.02 ± 0.00	0.02 ± 0.01	ND	ND
	Phenylethanol	3.99 ± 0.05	5.09 ± 0.05	5.23 ± 0.03	1.94 ± 0.39
	3-hydroxy-4-methoxytoluene	0.24 ± 0.02	0.21 ± 0.02	0.24 ± 0.01	ND
	Phenethyl isobutyrate	ND	0.06 ± 0.01	0.09 ± 0.00	ND
	DL-β-ethylphenylethyethanol	ND	0.05 ± 0.00	0.05 ± 0.01	ND
Aromatic compound	O-cresol	ND	0.01 ± 0.00	ND	ND
	Phenol	0.11 ± 0.00	0.16 ± 0.00	0.19 ± 0.03	2.10 ± 0.09
	4-ethyl guaiacol	0.46 ± 0.02	0.42 ± 0.03	0.48 ± 0.07	ND
	4-phenyl-3-butene-2-alcohol	0.05 ± 0.01	0.08 ± 0.00	ND	ND
	P-cresol	0.12 ± 0.01	0.33 ± 0.00	0.37 ± 0.00	ND
	P-ethylphenol	0.13 ± 0.02	0.16 ± 0.00	0.11 ± 0.02	ND
	5-vinyl-2-methoxy-phenol	0.03 ± 0.00	0.24 ± 0.00	0.11 ± 0.01	ND
	Ethyl 2-hydroxy-3-phenylpropionate	1.08 ± 0.03	1.33 ± 0.04	0.96 ± 0.13	0.18 ± 0.01
	Phenyl ethyl methoxyacetate	0.05 ± 0.00	0.07 ± 0.01	ND	ND
	4-vinylphenol	ND	0.04 ± 0.00	ND	ND
	Benzoic acid	0.09 ± 0.00	0.10 ± 0.00	0.11 ± 0.02	0.90 ± 0.02
	Diisobutyl phthalate	ND	0.02 ± 0.00	ND	ND
	Phenylacetic acid	0.03 ± 0.01	0.06 ± 0.03	ND	ND
	Ethyl dihydroferulate	0.09 ± 0.02	0.04 ± 0.01	ND	ND
	Caproate - 2-phenyl ester	ND	ND	ND	1.84 ± 0.18
	4-methylphenol	ND	ND	ND	35.77 ± 1.06

	3-methyl-2-phenyl-butyric acid	ND	ND	ND	0.23 ± 0.07
	1-methylethyl phenylpropionate	ND	ND	ND	0.52 ± 0.09
	2-methoxy-4-methylphenol	ND	ND	ND	0.89 ± 0.11
	Ethyl benzoate	ND	ND	ND	2.71 ± 0.31
	Propyl phenylacetate	ND	ND	ND	0.43 ± 0.03
	Furanyl ethyl ether	0.89 ± 0.02	1.27 ± 0.07	0.56 ± 0.05	ND
	2, 3-dimethyl-5-ethylpyrazine	0.05 ± 0.00	0.06 ± 0.01	0.15 ± 0.01	ND
	Furfural	ND	0.04 ± 0.00	0.08 ± 0.00	1.67 ± 0.23
	Ethyl 3-methylthiopropionate	0.38 ± 0.01	0.52 ± 0.01	0.73 ± 0.03	ND
	3-furanyl alcohol	0.57 ± 0.00	0.85 ± 0.01	1.09 ± 0.03	ND
Others	Hexanoic acid furfuryl ester	ND	ND	ND	1.04 ± 0.11
	2-acetyl pyrrole	0.04 ± 0.00	0.05 ± 0.01	0.05 ± 0.00	ND
	Coconut aldehyde	0.23 ± 0.02	0.26 ± 0.05	0.31 ± 0.04	ND
	Cypress brain	0.01 ± 0.00	0.02 ± 0.00	0.03 ± 0.01	ND
	2,5-Bis (methoxymethyl) furan	0.11 ± 0.02	0.11 ± 0.00	ND	ND

Note: ND, not detected.

Table S2. Number of volatile components in pit-mud and fermented grains of different depths in fermentation cellars of compound-flavor baijiu.

Kinds/Number	Different samples of fermented grains			Pit-mud
	CA	CM	CB	CP
Aldehydes and ketones	1	0	1	1
Alcohols	1	0	2	1
Acids	7	8	7	11
Esters	27	28	26	36
Aromatic compound	23	28	19	14
Others	8	9	8	2
Total	67	73	63	65
